# Impact of fatty degeneration on the functional outcomes of 38 patients undergoing surgical repair of gluteal tendon tears

**DOI:** 10.1007/s00402-021-03787-2

**Published:** 2021-03-02

**Authors:** Alexander Maslaris, Thomas P. Vail, Alan L. Zhang, Rina Patel, Stefano A. Bini

**Affiliations:** 1 Department of Orthopaedic Surgery, University of California, 400 Parnassus Avenue, MU320-W, San Francisco, CA 94143 USA; 2 Department of Orthopaedics, Friedrich-Schiller University of Jena, Campus Eisenberg, 07607 Eisenberg, Germany; 3 Department of Orthopaedics and Trauma Surgery, Alfried-Krupp Hospital, Campus Rüttenscheid, 45131 Essen, Germany; 4 Department of Radiology of Biomedical Imaging, University of California, 400 Parnassus Avenue, MU320-W, San Francisco, CA 94143 USA

**Keywords:** Gluteal tendon rupture, Gluteus medius et minimus tear, Gluteal tendon repair, Gluteal tendon refixation techniques, Fatty degeneration of gluteal muscles

## Abstract

**Background:**

Gluteal tendon tears (GTT) can cause pain and weakness of the hip. We analyze the impact of gluteal muscle fatty degeneration, atrophy and tear morphology on clinical outcomes of surgical repair.

**Methods:**

All sequential patients receiving surgical repair of GTTs via anchor sutures between 1/2015 and 11/2018 were retrospectively identified. MRIs were reviewed by a radiologist for tendon retraction, muscle atrophy and tear size. The Goutallier-Fuchs Classification (GFC) was used to quantify fatty degeneration as < 2° or ≥ 2°. Demographic and clinical variables were abstracted from the electronic records. The surveys HHS Section 1 and HOOS Jr. were obtained at last follow-up. The Pearson correlation and one-way ANOVA tests served for statistical analysis of clinical variance.

**Results:**

38 patients were identified, 29 (76.3%) were female. The average age was 67. Of the 11 (28.9%) patients with a prior hip arthroplasty 87.5% of primary THAs had a direct lateral approach. 29 (76.3%) patients were treated open and 9 (23.7%) arthroscopically. At an average follow-up of 20.9 months, patients reported a significant improvement in pain (97%), analgesic use (85.7%), limp (52.6%) and abduction strength (54.2%) (all: *P* ≤ 0.01). GFC ≥ 2° were associated with significantly worse outcomes in terms of limp (0.19/3 vs. 1.2/3, *P* = 0.05), HHS-S1 (58.19 vs. 71.68, *P* = 0.04) and complication rates (37.5% vs. 0%, *P* = 0.02). There was a strong correlation between tear retraction (*P* = 0.005), tear size (*P* = 0.009) and muscle atrophy (*P* = 0.001) with GFC ≥ 2° but not with clinical outcomes. GFC ≥ 2° was strongly related to lateral THA exposures (*P* < 0.001). Surgical approach had no impact on clinical outcomes.

**Conclusion:**

While fatty degeneration can negatively impact functional outcomes, pain relief is reliably achieved. Tear morphology and muscle atrophy did not correlate with outcomes in this patient cohort. Patients should be counseled to expect a residual limp after surgery if they have GFC ≥ 2° on MRI.

## Introduction

Gluteal tendon tears (GTT) are often unrecognized and treated inadequately [1]. Depending on their severity and etiology, symptoms may vary from a sole lateral hip pain to a persistent abductor insufficiency of the hip. Structural parameters of GTT such as tear size, retraction of the torn tendon [2–4], fatty degeneration and atrophy of the injured muscle [3, 5–7] may impact the clinical outcomes of surgical treatment. Demographic aspects of the individual patient like age [8, 9], gender [10], bone mineral density (BMD) [11, 12] and body mass index (BMI) as well as the presence of a preexisting total hip arthroplasty (THA) and the number of previous revision THAs (RHA) [13] pose potential risk factors for the success of the initiated therapy. In particular, postsurgical GTTs are associated with the direct lateral THA approach [14, 15] or even the anterolateral THA approach [16].

The surgical treatment of GTT includes the reattachment of the torn tendon using sutures/suture anchors in single or double-rows with or without fixation via various augmentation techniques depending on the severity of the GTT and the soft tissue quality [2, 11, 17–23]. Some authors have recommended muscle transfer techniques as the only solution in cases of non-reconstructable GTTs with severe soft tissue damage [24–33].

Available evidence suggests that surgical repair of GTT with either open [2, 5, 17, 18, 20–22, 34–45] or endoscopic techniques [6, 46–53] can lead to very good to excellent results with a significant improvement in pain. However, risk factors such as fatty degeneration (FD), muscle atrophy (MA) and tear morphology (TM) may impact negatively the surgical results. Thus, more complex tears and patients with higher comorbidities tend to show less favorable outcomes and needed commonly to be treated in an open fashion [54].

Related literature up to now suffers from inhomogeneity and small sample sizes making reliable conclusions and developing transparent treatment algorithms impossible. Thus, little is known about the comparative impact of FD, MA and TM on the postoperative outcomes between open (OGR) and endoscopic gluteal repair (EGR) in minimizing pain and restoring function.

The aim of the study is to analyze the impact of fatty degeneration and the other risk factors on the mid-term results in a large cohort of patients who underwent either EGR or OGR of GTTs via suture anchors.

## Materials and methods

Approval from the local Institutional Review Boards was granted. A retrospective single-center cohort study from a university hospital was conducted. All sequential patients receiving surgical repair of GTTs via anchor sutures between 1/2015 and 11/2018 were identified and retrospectively recruited for the study. Treatment was divided in: (1) EGR, (2) primary OGR (pOGR) and (3) revision total hip arthroplasties with OGR (rOGR).

All patients who were included in the study presented with persistent lateral hip pain with or without signs of hip abductor insufficiency and a positive magnetic resonance imaging (MRI) for GTT prior to surgery. Exclusion criteria were septic surgeries, active tumors or known neurological diseases that affected the hip, no preoperative MRI or inability to assess due to massive hardware artifacts, patients who refused to participate to the study and finally cases with missing or inconsistent documentation.

Demographic and clinical variables were abstracted from the electronic record using an institutional database. Age, gender, BMI and comorbidity level, as defined by the America Society of Anesthesiologist (ASA), were collected.

The GTT etiology was divided into (1) degenerative, (2) posttraumatic or (3) postoperative (prior THA) causes.

Preoperative x-rays were assessed to measure the femoral offset and look for any malposition or instability of preexisting implants on the hip or lumbar spine.

The preoperative MRI was used to evaluate the fatty infiltration and atrophy of the gluteal muscles and to analyze the complexity of the GTTs.

**Fig. 1:**
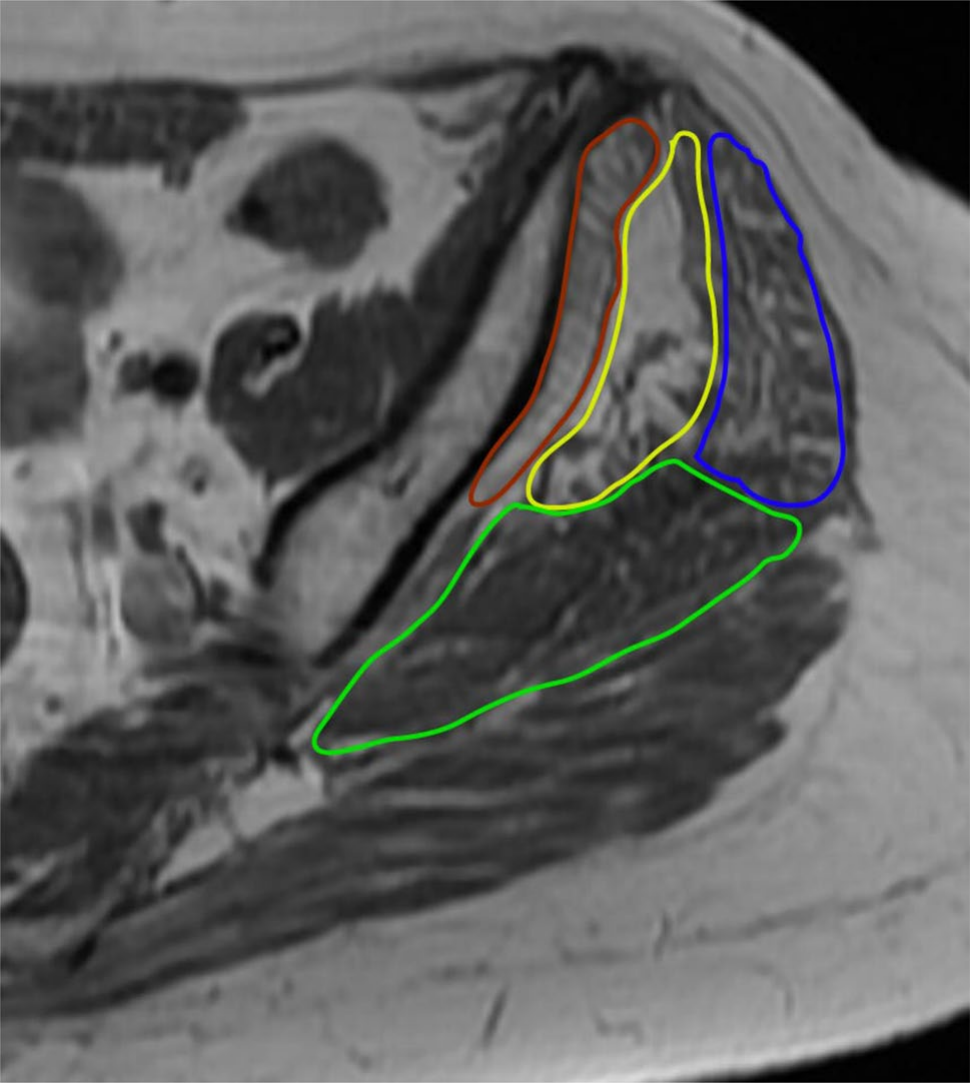
An axial T1 sequence of an MRI illustrating the Gmin and the three district parts of Gmed, that were included in the assessment of fatty degeneration as described by Thaunat [6]: Gmin (rot), anterior portion (blue), mid portion (yellow) and posterior portion of Gmed (green)

The fatty infiltration of the gluteal muscles was grouped based on the Goutallier-Fuchs classification (GFC) in: 0 = normal muscle, 1 = muscles with some fatty streaks, 2 = muscles with moderate fatty streaks (more muscle than fat), 3 = muscles with severe fatty streaks (equal amounts of fat and muscle), and 4 = muscles with more fat than muscle [55–57]. Based on recent studies [3, 5], GFC was further divided into two clinically relevant main groups, namely into below (GFC < 2°) and above grade 2 (GFC ≥ 2°). The assessment included the gluteus minimus (Gmin) and three distinct parts of the gluteus medius muscle (Gmed): the anterior, mid and posterior portion of Gmed (Fig. 1). This technique is described by Thaunat et al. [6]. According to that study, gluteus medius and minimus muscles were evaluated on the axial T1 sequences without fat saturation on the first axial slice inferior to the sacroiliac joint.

Muscle atrophy of the hip abductors was defined as the reduction of the size of its components by ≥ 25% then that of the contralateral side [58].

**Fig. 2:**
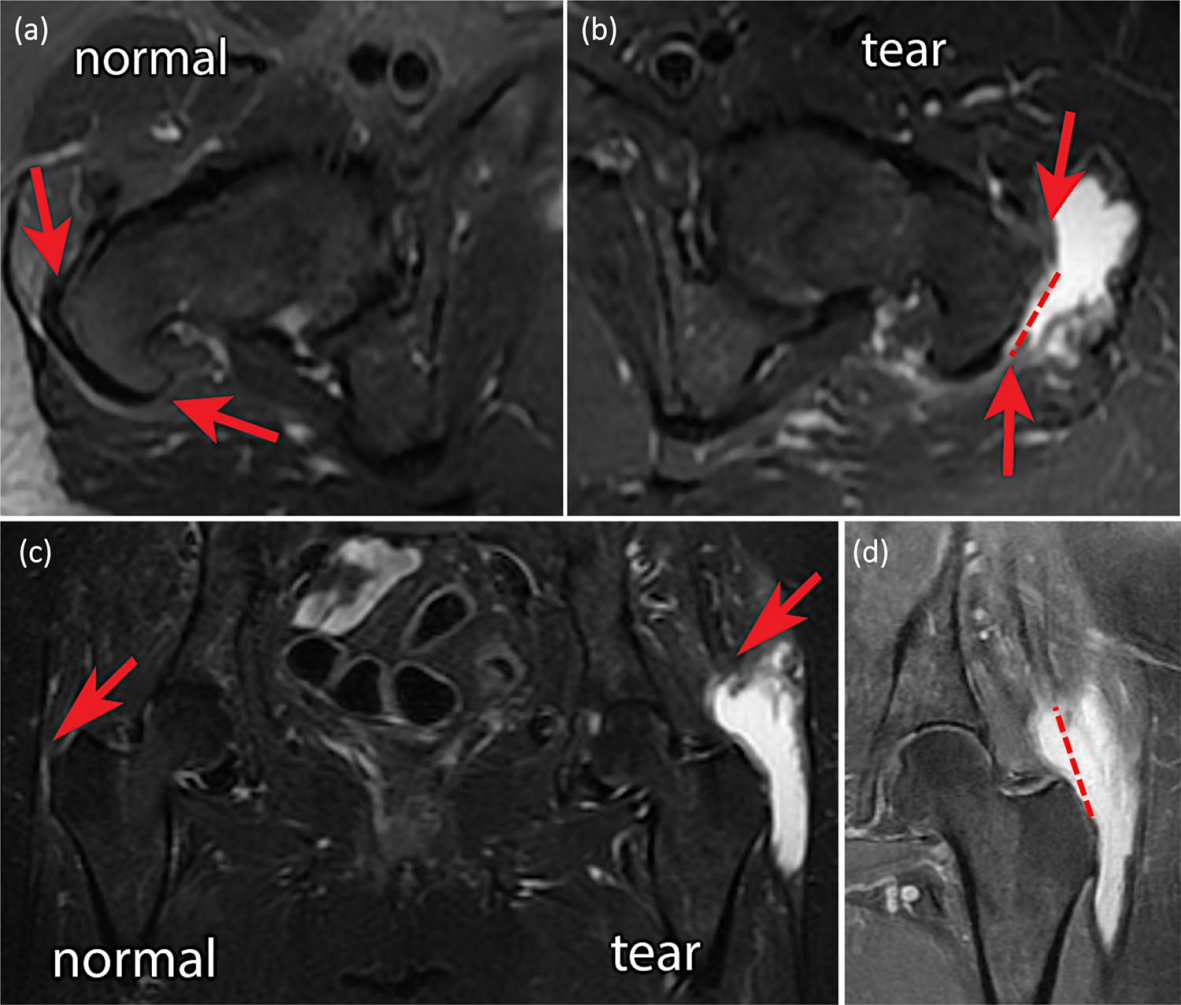
**a–d** Preoperative MRI revealing a GTT on the left side. In the axial views **a** the right healthy side and **b** the anteroposterior detachment on the lateral aspect of trochanter. In the coronal plane **c** and **d** the retraction of the torn gluteal muscle compared to the healthy contralateral hip

The gluteus medius and minimus tendons were assessed separately for presence or absence of tear. All available fluid-sensitive sequences (STIR or T2 fat saturation) were used to evaluate and characterize the tear. The tears were characterized as full-thickness lesions (FT) or partial-thickness lesions (PT), which were further differentiated into lateral bursal-side (LPT) or medial joint-side partial tears (MPT). The length of tendon retraction was measured from their attachment on the greater tuberosity. The anteroposterior width of the tear was also measured (Fig. 2a–d). Any evidence of bursitis was classified in 0 = none, 1 = mild, 2 = moderate, or 3 = severe as described by Chi et al. [59]. All MRI measurements mentioned above were performed by an experienced musculoskeletal radiologist.

All patients included in the study were contacted postoperatively via e-mail or phone and invited to an anonymous survey to evaluate the postoperative results. For this purpose, following patient-reported outcome measures (PROMs) were used: The Hip disability and Osteoarthritis Outcome Score Junior (HOOS Jr.) [60], the questionnaire of the first section of Harris Hip Score (HHS-S1) [61], that concerns pain and function features, and the Visual Analog Scale of pain (VAS). Furthermore, the amount and type of analgesics been taken at the time of the survey due to still existing hip pain was verified.

Following postoperative outcome values were analyzed:

1. Pain intensity, according to VAS scale
2. Limp severity based on the HHS protocol, classified as: 0 = non, 1 = mild, 2 = moderate, 3 = severe and 4 = extreme
3. Hip abduction strength, according to the Medical Research Council Score (MRC-Score) [62]
4. Number of non-opioid analgesics including nonsteroidal anti-inflammatory drugs (NSAIDs) and analgesic adjuvants (Acetaminophen, Ibuprofen, Naproxen, Meloxican, Ketorolac, Celecoxib, Gabapentin, Temazepam, Metaxalone, Baclofen)
5. Number of opioid drugs (Hydrocodone, Oxycodone, Tramadol) taken at the time of last follow-up.

The software IBM SPSS (Version 25.0) was used to perform statistical analysis. The Pearson correlation test was used to assess possible correlations between potential risk factors and outcomes. The two-tailed paired T-test was used to compare pairwise the postoperative results with the preoperative findings. The one-way analysis of variance (ANOVA) and the least significant difference (LSD) tests were performed for single or multiple intergroup comparisons between different patient subgroups.

## Results

From the 38 patients, who met inclusion criteria, 29 (76.3%) were females and the average age was 66.7. Eleven patients (29%) had a prior hip replacement, 87.5% of which were performed via a direct lateral approach.

29 (76.3%) patients were treated open and 9 (23.7%) endoscopically. In all cases, suture anchors were used for the repair of the GTTs (in 76% of cases: Suture Anchor Corkscrew 5.0 mm with #2 Fiberwire, Arthrex, Naples, FL, USA; in 16%: Nanotack Suture Anchor System with Iconix #2, Stryker, Kalamazoo, MI, USA; in 5%: PEEK Zip Suture Anchor 5.5 mm Stryker, Kalamazoo, MI, USA; in 3%: a GII Quickanchor with Orthocord, DePuy Mitek, Raynham, MA, USA). In two cases with advanced fatty degeneration, additional augmentation via nonabsorbable synthetic Mersilene mesh (GFC 2.7°) and Achilles tendon allograft fixed with 3.5 mm cortical screws (GFC 2.6°) were required.

**Fig. 3:**
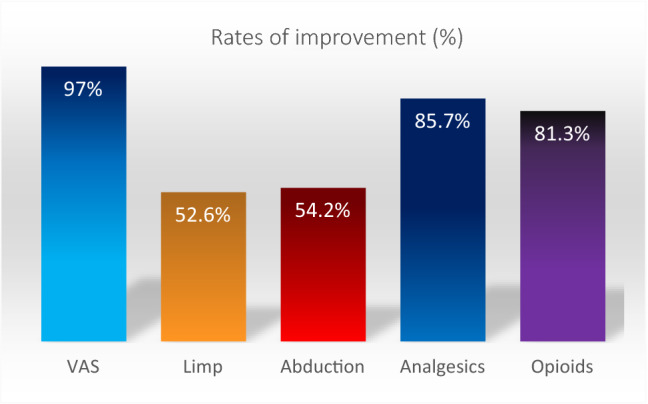
Percentage of the overall cases with verified improvement of the main outcome objectives after surgical treatment of GTT

At an average follow-up of 21 months, a significant overall improvement in pain (97%), limp (52.6%), and abduction strength (54.2%) as well as in reduction of non-opioid analgesic use (85.7%) and opioid use (81.3%) was noticed independent of surgical approach (all with *P* ≤ 0.01) (Fig. 3).

The differences between each GFC groups are summarized as follows (Table 1).

**Table 1:** Demographics, findings and outcomes in relation to fatty degeneration of gluteal muscle according to Goutallier-Fuchs Classification (values in means + standard deviation)

GFC	0°	1°	2°	3°	All
Nr	6 (15.8%)	16 (42.1%)	10 (26.3%)	6 (15.8%)	38
Age	64 ± 12.2	63 ± 8.6	69,5 ± 11.3	71,5 ± 4.6	66.7 ± 9.9
Gender (females)	5 (83.3%)	13 (81.3%)	6 (60%) females	5 (83.3%)	29 (78%)
ASA	1 (16.7%) I° 3 (50%) II° 2 (33.3%) III°	0 (0%) I° 14 (87.5%) II° 2 (12.5%) III°	1 (10%) I° 7 (70%) II° 2 (20%) III°	1(16.7%) I° 4 (66.7%) II° 1 (16.7%) III°	3 (7.9%) I° 28 (73.7%) II° 7 (18.4%) III°
BMI	28.24 ± 6.5	27,7 ± 4.2	30,2 ± 7.6	27 ± 5.7	28,33
Etiology	5 (83.3%) spontaneous 1 (17%) posttraumatic	13 (81.3%) spontaneous 2 (12,5%) posttraumatic 1 (6.3%) prior THA	2 (20%) spontaneous 1 (10%) posttraumatic 7 (70%) prior THA *4 primary THA* *3 revision THA*	2 (33.3%) spontaneous 1 (16.7%) posttraumatic 3 (50%) prior THA	22 (57.9%) spontaneous 5 (13.2%) posttraumatic 11 (28.9%) prior THA
THA approaches	None	1/1 LA	4/4 LA	2/3 LA	7/8 (87.5%) LA
Prior RHA nr	None	None	2 (20%) ≥ 3 RHA 1 (10%) < 3 RHA	none	3 prior RHA (1: < 3, 2: ≥ 3 RHAs)
FOS (mm)	46.5 ± 1.5	46.8 ± 6.9	48.2 ± 4.7	46.6 ± 6.7	47.1 ± 5.6
Injured tendons	60% Gmed 20% Gmin 20% Gmed & Gmin	50% Gmed 12.5% Gmin 31.3% Gmed & Gmin	50% Gmed 0% Gmin 50% Gmed & Gmin	16.7% Gmed 83.3% Gmed & Gmin	17 45.6% Gmed 3 8.1% Gmin 16 43.2% Gmed & Gmin
Tear type Gmed	40% PT 40% FT	31.3% PT 43.8% FT	40% PT 60% FT	16.7% PT 83.3% FT	31.6% PT 52.6% FT
Tear type Gmin	0% FT 40% PT	31.3% PT 18.8% FT	30% PT 20% FT	33.3% PT 50% FT	26.3% PT 21% FT
Retraction Gmed in mm	0	5.6 ± 10.7	18.7 ± 18.3	20.5 ± 23.9	8.8 ± 1.5
Tear size in mm	11.3 ± 2.3	13.8 ± 8.9	19 ± 11.1	30.2 ± 9.9	15.5 ± 10.8
Muscle atrophy (< 25%)	50%	54%	94%	100%	75%
Bursitis	1.2 ± 0.8	1.1 ± 0.8	1.9 ± 1.1	2.3 ± 1.2	1.54 ± 1.0
Treatment	5 (83.3%) OGR 1 (16.7%) EGR	9 (56.3%) OGR: *8 (50%) pOGR* *1 (6.3%) rOGR* 7 (43.8%) EGR	9 (90%) OGR: *3 (30%) pOGR* *6 (60%) rOGR* 1 (10%) EGR	6 (100%) OGR: *3 (50%) pOGR* *3 (50%) rOGR*	29 (76.3%) OGR: *19 (50%) pOGR* *10 (26.3%) rOGR* 9 (23.7%) EGR
Technique	10/6 anchors (mean 1.7 + 0.5)	31/16 anchors (mean 1.9 ± 0.9)	29/10 anchors (mean 2.9 ± 1.1) 1/10 mesh 1/10 GmaxT	16/6 anchors (mean 2.7 ± 1.2)	86/38 anchors (mean 2.3 ± 1.0) 1/38 mesh
Follow-up (months)	24.7 ± 10.5	22.4 ± 16	19.7 ± 5.5	15.3 ± 10.5	20.92 ± 12.51
Abduction av. Improvement	+ 0.5 ± 0.6/5	+ 0.46 ± 0.9/5	+ 0.4 ± 0.5/5	+ 0.5 ± 0.5/5	+ 0.46 ± 0.7/5**
Limp av. improvement	− 1.2 ± 0.8/3	− 1.1 ± 1.1/3	0 ± 1.2/3	− 0.5 ± 1.8/3	− 0.68 ± 1.3/3**
Non-opioid analgesics %-reduction	− 7/8 (87.5%)	− 20/25 (80%)	− 16/20 (80%)	− 7/7 (100%)	− 50/60 (83.3%)**
Opioid ‘%-reduction	− 2/2 (100%)	− 6/8 (75%)	− 3/4 (75%)	0.0	− 11/14 (78.6%)**
VAS av. improvement	− 3.6/10	− 4.9/10	− 3.7/10	− 3.5/10	− 4.14/10 **
*HOOS Jr*	67 ± 17.4/100	78.7 ± 14.3/100	67 ± 29.5/100	74.6 ± 18.4/100	72.99 ± 20.6/100
*HHS Sect. 1 (pain/function)*	65.2 ± 11.3/91	74 ± 14.4/91	58 ± 21.3/91	58.5 ± 24.3/91	65.51 ± 18.9/91
Complications	None	None	4 × after rOGR	2 × after rOGR	6/38 (16%) all after rOGR

*GFC* Goutallier-Fuchs Classification, *ASA* American Society of Anesthesiologist, *BMI* Body-Mass-Index, *THA* total hip arthroplasty, *LA* lateral approach*, RHA* revision hip arthroplasty, *OGR* open gluteal repair, *pOGR* primary open gluteal repair, *rOGR* revision hip arthroplasty with open gluteal repair, *EGR* endoscopic gluteal repair, *GmaxT* gluteus maximus transfer, *Gmed* gluteus medius, *Gmin* gluteus minimus, *PT* partial tear, *FT* full-thickness tear, *VAS* Visual Analog Scale of pain, *HOOS* Hip disability and Osteoarthritis Outcome Score Junior, *HHS* Harris Hip Score

*Significance at the 0.05 level (2-tailed)

**Significance at the 0.01 level (2-tailed)

**Fig. 4:**
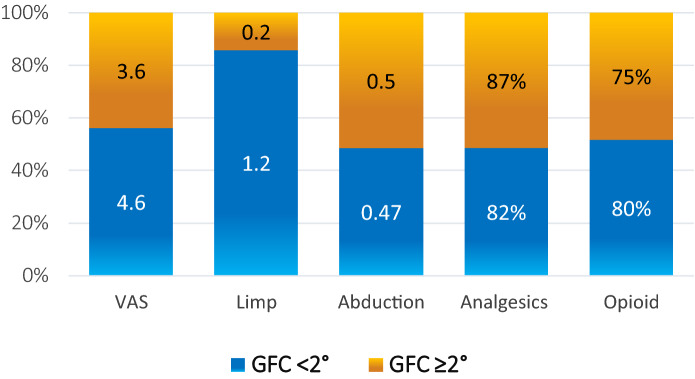
Means of postoperative improvement of the tested outcome variables between GFC < 2° and GFC ≥ 2°

Whilst GFC < 2° had no negative impact on clinical outcomes, GFC ≥ 2° was associated with significantly worse outcomes with respect to a patient’s reported limp (average: 0.19/3 vs. 1.2/3, *P* = 0.05) (Fig. 4), HHS-S1 scores (58.19 vs. 71.68, *P* = 0.04) and complication rates (37.5% vs. 0%, *P* = 0.02) (Tables 2 and 3). Though GTT retraction (*P* = 0.005), tear size (*P* = 0.009) and significant association with the postoperative clinical outcome values. Cases with GFC ≥ 2° were strongly correlated with THA lateral exposures (*P* < 0.001) and the need to use more anchors for the GTT repair (*P* = 0.004). The severity of trochanteric bursitis on pre-operative MRI was significantly associated with both the fatty degeneration and atrophy of the injured gluteal muscles (*P* = 0.02 and *P* = 0.008, respectively). 83.8% of all GTT revealed a mean bursitis scale of 1.54 ± 1.0°.

**Table 2:** Mean differences of pain, limp, abduction strength, Nr. of analgesics and opioids before and after surgery for the groups GFC < 2° and GFC ≥ 2°

	VAS	Limp	Abduction	Analgesics	Opioid
GFC < 2°	− 4.6**	− 1.2**	+ 0.47*	− 82%**	− 80%**
GFC ≥ 2°	− 3.6**	− 0.2	+ 0.5*	− 87%**	− 75%
All patients	− 4.14**	− 0.74**	+ 0.46**	− 85.7%**	78.6%**
One-way ANOVA of mean differences comparing outcomes of independent groups					
GFC < 2° vs. ≥ 2°	0.95	0.98*	0.03	5%	5%

*Significance at the 0.05 level (2-tailed)

**Significance at the 0.01 level (2-tailed)

**Table 3:** Demographics, findings and outcomes in relation to fatty degeneration of the muscle of gluteus medius et minimus according to Goutallier-Fuchs Classification divided in < 2° and ≥ 2° with reports of its significant differences (*P* values)

GFC	< 2°	≥ 2°	< 2° vs. ≥ 2° *P* values
Nr	22 (57.9%)	16 (42.1%)	0.33
Age	63 ± 9.4	70 ± 9.2	**0.03**
Gender (females)	18 (81.8%)	11 (68.8%)	0.38
BMI	27.81 ± 4.7	29 ± 6.9	0.56
Etiology	18 (81.8%) spontaneous 3 (13.6%) posttraumatic 1 (4.5%) prior THA	4 (25%) spontaneous 2 (12.5%) posttraumatic 10 (62.5%) prior THA	** < 0.001**
Lateral THA approaches	1/1 (100%) LA	6/7 (85.7%) LA	**0.02**
Prior RHA (nr.)	None	2/10 (20%) ≥ 3 RHA 1/10 (10%) < 3 RHA	
Injured tendons	52.4% Gmed 14.3% Gmin 28.6% Gmed & Gmin	37.5% Gmed 0% Gmin 62.5% Gmed & Gmin	0.08
Tear type Gmed	38% PT 42.9% FT 19% isolated Gmin PT	31.3% PT 68.8% FT	**0.03**
Tear size in mm	13.4 ± 8.1	23.3 ± 11.7	**0.02**
Retraction Gmed in mm	3.2 ± 8.4	19.4 ± 19.3	**0.02**
Muscle atrophy (≥ 25%)	53%	96.4%	**0.002**
Bursitis	1.14 ± 0.8	2.1 ± 1.1	**0.01**
Treatment	14 (63.6%) OGR: *13 (59.1%) pPOGR* *1 (4.5%) rOGR* 8 (36.4%) EGR	15 (93.8%) OGR: *5 (31.3%) pOGR* *10 (62.5%) rOGR* 1 (6.3%) EGR	** < 0.001**
Nr. of anchors	41/22 (mean 1.86 ± 0.8)	45/16 (mean 2.81 ± 1.1)	**0.01**
Abduction av. Improvement	+ 0.47 ± 0.8/5	+ 0.5 ± 0.5/5	0.91
Limp av. improvement	− 1.2 ± 1/3	− 0.2 ± 1.4/3	**0.05**
Analgesics av. reduction	− 27/33 (-81.8%)	− 23.5/27 (− 87%)	0.31
Opioid av. reduction	− 8/10 (− 80%)	− 3/4 (− 75%)	0.35
VAS av. improvement	− 4.6 ± 2.6/10	− 3.6 ± 2.9/10	0.32
*HOOS Jr*	75.6 ± 15.6/100	69.7 ± 25.4/100	0.44
*HHS Section 1* *(pain & function)*	71.7 ± 13.9/91	58.2 ± 21.7/91	**0.04**
Complications	None (0%)	6 after ROR (37.5%)	**0.02**

Significant values are highlighted in bold

*GFC *Goutallier-Fuchs Classification, *BMI *Body-Mass-Index, *THA* total hip arthroplasty, *LA* lateral approach, *RHA *revision hip arthroplasty, *Gmed *gluteus medius, *Gmin *gluteus minimus, *PT *partial tear, *FT *full-thickness tear, *OGR *open gluteal repair, *pOGR *primary open gluteal repair, *rOGR *revision open gluteal repair (revision total hip arthroplasty with open repair), *EGR *endoscopic gluteal repair

From the 28 preoperative MRIs with possible evaluation of muscle atrophy, 24 (85.7%) cases revealed a MA on both the Gmed and Gmin whereas in 18 (64.3%) of the cases only the Gmed was affected.

Muscle atrophy was associated with the etiology of a GTT (*P* = 0.039), being evidenced more often in the rOGR group. Atrophy was also correlated with the Gmed retraction (*P* = 0.035) and tear size (*P* = 0.037). GTTs with a MA ≥ 25% showed significantly higher levels of Gmed retraction (0 mm vs. 15.3 ± 1.9 mm, *P* = 0.01) and fatty degeneration (1.48 ± 0.9 vs. 2.6 ± 0.8, *P* < 0.001) and were found more often in cases with preexisting THA (50% vs. 10%, *P* = 0.02) when compared to the GTT group without MA (Table 4). However, MA did not have any significant impact on clinical outcomes and functional PROMs at the last postoperative follow-up.

**Table 4:** Demographics, findings and outcomes in relation to atrophy of the abductor muscle with reports of significant differences (*P* values)

Atrophy of abductor muscles (≥ 25%)	No	Yes	< 25% vs. ≥ 25% *P* values
Cases (%)	36%	64%	
Age	61.9 ± 9.2	68 ± 9.6	0.12
Gender (females)	7 (70%)	15 (83.3%)	0.46
BMI	28.29 ± 4.7	28.28 ± 6.8	1.00
ASA	0 (0%) I° 10 (100%) II° 0 (0%) III°	2 (11.1%) I° 11 (61.1%) II° 5 (27.8%) III°	0.27
Etiology	8 (80%) spontaneous 1 (10%) posttraumatic 1 (10%) prior THA	7 (38.9%) spontaneous 2 (11.1%) posttraumatic 9 (50%) prior THA	**0.02**
Lateral THA approaches	1/1 LA	6/9 LA	0.14
Prior RHA (nr.)	None	2/9 (22.2%) ≥ 3 RHA 1/9 (11.1%) < 3 RHA	0.10
Injured tendons	50% Gmed 20% Gmin 30% Gmed & Gmin	50% Gmed 0% Gmin 50% Gmed & Gmin	0.60
Tear type Gmed	50% PT 30% FT	33.3% PT 66.7% FT	**0.05**
Tear size in mm	13.7 ± 8.2	20 ± 1.3	0.17
Retraction Gmed in mm	0 ± 0	15.3 ± 1.9	**0.01**
GFC	1.48 ± 0.9	2.6 ± 0.8	** ≤ 0.00**
Bursitis	0.8 ± 0.6	2.0 ± 1.1	** ≤ 0.00**
Treatment	4 (40%) OGR: *3 (30%) pOGR* *1 (10%) rOGR* 6 (60%) EGR	16 (88.9%) OGR: *7 (38.9%) pOGR* *9 (50%) rOGR* 2 (11.1%) EGR	**0.02**
Nr. of anchors	22/10 (mean 2.2 ± 0.9)	45/18 (mean 2.5 ± 1.2)	0.46
Abduction av. improvement	+ 0.22 ± 0.4/5	+ 0.61 ± 0.5/5	0.09
Limp av. improvement	− 0.9 ± 1.2/3	− 0.4 ± 1.5/3	0.38
Analgesics av. reduction	− 12/14 (− 85.7%)	− 28/31 (-90.3%)	0.19
Opioid av. reduction	− 4/5 (− 80%)	− 4/5 (− 80%)	0.40
VAS av. improvement	− 3.9 ± 2.0/10	− 3.8 ± 2.6/10	0.97
*HOOS Jr*	72.1 ± 17.36/100	75.6 ± 15.6/100	0.71
*HHS Section1* *(pain & function)*	72.88 ± 14.3/91	63.17 ± 21.12/91	0.19
Complications	1 (10%) after rOGR	4 (22%) all after rOGR	0.89

Significant values are highlighted in bold

## Discussion

Independent of the operative approach used to treat gluteal tendon tears, fatty degeneration of gluteal muscles GFC ≥ 2° seems to be associated with significantly worst functional outcomes after surgical repair. GTT muscle atrophy and retraction did not reveal any association to the postoperative results in our patient cohort. Nevertheless, this should be considered cautiously since it might be due to the small subgroup sizes that met inclusion criteria within the current study design. Finally, statistically significant improvement of all main outcome measures (VAS, limp, abduction, analgesics, opioids) were achieved in all cases of GTT after surgical treatment (Tables 1 and 2) suggesting that even patients with advanced fatty degeneration may benefit from direct surgical repair.

The subgroup analysis showed detailed impact of fatty degeneration. GFC ≥ 2° was associated with considerably inferior outcomes in the functional section S1 of HHS and limp after GTT surgical repair when compared to GFC < 2° (*P* = 0.04 and *P* = 0.025, respectively). Furthermore, complication rates were significantly higher in the GFC ≥ 2° group (37.5% vs. 0%, *P* = 0.02). However, whilst it is known that fatty degeneration has a negative impact on postoperative functional outcomes [3, 6, 7], our study suggests that pain relief can be reliably accomplished after surgical treatment of GTTs regardless of the degree of GFC. Thus, our results align with the available literature, suggesting that surgical reattachment of GTT is an effective treatment option for persistent lateral hip pain [44, 63].

Current data indicates that endoscopic and open surgical approaches are both equally successful [54]. However, there are some fundamental differences between these two approaches to consider when choosing the proper treatment strategy: endoscopic approach is better suited to smaller tears and tears of the gluteus minimus (cite our prior paper here) and has the benefit of also being able to address articular-side pathologies during the same procedure. Most arthroscopic procedures were used in patients with a low to moderate degree of fatty degeneration (GFC < 2°). There are no data available to our knowledge assessing the efficacy of endoscopic GTT repair in cases with severe GFC. EGR also requires specialized equipment and a considerable learning-curve with the risk of poor anchor placement [64]. Open approaches offer a greater exposure and access to the retracted edge of chronic GTT and therefore provide greater treatment flexibility with more options for fixation including, if required, the use of augmentation or reconstruction techniques in cases with more extended soft tissue damage [24–33, 65, 66] or palsy of the superior gluteal nerve [16, 67, 68].

In the current study, MRI findings prior to surgery revealed a strong correlation between fatty degeneration and other abnormal structural properties of the GTTs such as the retraction (*P* = 0.005), tear size (*P* = 0.009), muscle atrophy (*P* = 0.001) and the severity of trochanteric bursitis (*P* = 0.02). There was also a significant difference in the severity of retraction, tear size, muscle atrophy and bursitis of the GTTs between GFC < 2° and GFC ≥ 2° (Table 3). However, we could not find any significant impact of the above-mentioned variables on the postoperative results after GTT repair. In contrast, previous publications support the importance of tear size and retraction on the success of surgical treatment [2, 3]. Amstutz and Maki using the trochanteric approach for THAs in the late 70′s found that post-operative abductor insufficiency was associated with pre-operative separation gaps > 2 cm [69]. In a recent study on 46 RHA’s, Caviglia et al. drew the same conclusion indicating significantly worst hip abduction when GTT trochanteric displacement was > 2.5 cm. This finding was independent of the tear type between partial and full-thickness lesions [2].

Makridis et al. in a relative large patient cohort (70 hips) reported that muscle atrophy and not FD had a negative impact on functional outcomes (*P* = 0.05) after open double-row GTT repair [5]. However, the majority of the patients included were healthy individuals with no history of THA, trauma, or any systemic inflammatory disease. 94% of these cases had partial lesions of the anterior portion of the Gmed and 20% (*n* = 14) a FD ≥ 2°. In 60% of their cases there was neither FD nor MA evidenced. In our cohort, FD ≥ 2° was found in 42.1% and MA in 64%, while cases with FD ≥ 2° were associated with MA in 96.4% (Tables 3 and 4).

Based on known similarities between GTTs and rotator cuff tears (RCT) [70] and the available data originating from the shoulder surgery literature, duration of symptoms [71] and age of the patient [72] seem to be associated with increased fatty degeneration of the RCT muscles, whereas, inactivity and nerve injuries lead to muscle atrophy [73].

Total hip arthroplasty (THA) is considered one of the most successful and effective orthopedic procedures. However, 6% of the patients remain unsatisfied due to persistent pain after surgery [74, 75]. Limited evidence exists concerning the exact etiology of pain in this critical patient group. The surgical technique chosen to perform a THA may play an essential role in pain generation if it leads to damage to the abductor tendons. A high incidence of postoperative Trendelenburg sign (27.6%) and limp (4–20%) has been described after use of the lateral THA approach [14, 15, 19, 76].

Thus, ongoing lateral hip pain with Trendelenburg gait in a patient with a history of a direct or antero-lateral THA should always raise suspicions of a GTT or failed repair of the partial Gluteus Medius take-down. Spontaneous partial GTTs are most commonly found on the anterior portion of the Gmed due to its thinner anterolateral layer [77].

The limitations of this work include the retrospective nature of the study and thus, the potential recall bias for some subjective measures of the PROMs that were collected and the resulting small sample size of the subgroups tested here, that might have an influence on the power of the results.

However, this is one of the largest descriptive studies that analyzes the impact of some clinically relevant structural properties of GTTs on the mid-term outcomes after surgical treatment. Our thorough MRI analysis of the GTTs by specialized musculoskeletal radiologist provides a clear and well-established methodology for classifying and reporting results from GTT repairs. Lastly, the majority of the repairs described in our study were performed on “massive” GTTs and outlines the results that can be expected with these patients. We hope that the current study provides important information that could guide physicians to be more aware of the potential benefits of GTT repairs and their early referral for surgical intervention before fatty atrophy sets in.

## Conclusion

We report overall excellent results in a large series of GTT repairs and a classification system to predict clinical outcomes from pre-operative imaging. While fatty degeneration can negatively impact functional outcomes, pain relief is reliably achieved. Tear morphology and muscle atrophy did not correlate PROMs in our cohort. Patients should be counseled to expect a residual limp after surgery if they have GFC ≥ 2° on MRI.
